# Serum myokines as potential biomarkers of myostatin inhibition in sport doping: a preliminary study on their baseline levels in elite athletes

**DOI:** 10.5114/biolsport.2024.132982

**Published:** 2023-11-17

**Authors:** Francesco Donati, Giorgia Morgan Biasini, Xavier de la Torre, Francesco Botrè

**Affiliations:** 1 Laboratorio Antidoping, Federazione Medico Sportiva Italiana, Rome, Italy; 2 REDs – Research and Expertise in anti-Doping sciences, ISSUL – Institute of sport sciences, University of Lausanne, Lausanne Switzerland

**Keywords:** Doping Biomarkers, Flow Cytometry Bead Array, Myokines, Myostatin, Myostatin Inhibition

## Abstract

We considered in this study the possibility of developing an indirect procedure for detecting myostatin inhibition/suppression, a practice that is prohibited as doping in sport. We have specifically considered the potential diagnostic utility of human serum myokines as indirect markers of myostatin inhibition. Myostatin, its main antagonist follistatin, and other myokines (follistatin-like 1, musclin, oncostatin, osteonectin, irisin, brain derived neurotrophic factor, and insulin-like growth factor-1) were selected as a panel of potential biomarkers whose levels may be altered following myostatine suppression. The serum levels of myostatin and of the nine myokines were measured in elite athletes of different age, sex, and sport discipline, and their cross correlation assessed by multivariate analysis. All myokines resulted to be measurable in human serum, except for musclin and irisine, whose levels were below the limits of quantitation in a reduced number of samples. Serum concentrations varied of different orders in magnitude (musclin and osteonectin < 1 ng/mL; follistatin, myostatine and irisine 1–5 ng/mL; brainderived neurotrophic factor, follistatin-like 1 and iinsulin-like growth factor-1 > 10 ng/mL), while no significant differences were found between female and male subjects, with the exceptions of follistatin-like 1 and musclin, showing a higher concentrations in females (p < 0.05). Levels of insulin-like growth factor 1 and brain derived neurotrophic factor were significantly higher in power athletes than in endurance ones. Multivariate statistics showed that musclin, follistatin-like 1 and oncostatin are more clustered and correlated to myostatin than other myokines, suggesting they could be considered as potential biomarkers of doping by myostatin inhibitors.

## INTRODUCTION

Myokines are a class of small proteins mainly produced and released from skeletal muscle cells and strictly involved in myogenesis, muscle contraction, and exercise-associated metabolic changes. In general, myokines play a significant role in various physiological functions, including muscle growth and metabolic homeostasis (reviewed in [1]). Myostatin (MYO), also known as Growth Differentiation Factor-8 (GDF-8), is a 25 kDa myokine belonging to the Transforming Growth Factor beta (TGF-β) family. MYO is expressed in skeletal muscles, acting as the key negative regulator of skeletal muscle cell growth and differentiation [2]. MYO specifically acts on muscle cells with the dual action of inhibiting the proliferation and differentiation of the myoblasts. Recent experimental evidence has revealed that muscles of knock-out mice for the MYO gene resulted to be two to three times larger than the wild-type ones. Transgenic mice expressed high levels of the MYO propeptide, and/or high levels of follistatin (FOL), which is the main natural antagonist of MYO, were expressed. The results of these experiments showed an increase in muscle similar to what had been observed in knockout mice for the MYO gene [3].

In recent years, great relevance has been devoted to these proteins in the processes of communication with other organs, such as liver and adipose tissue. However, since the main role of MYO is the inhibition of muscle growth, it is possible to obtain a condition of hypertrophy by antagonizing MYO in several ways. Substances or factors that can inhibit or suppress MYO could be therefore appealing for doping purposes, allowing to increase muscle mass, with an enhancement in terms of strength and, consequently, of the athletic performance.

Enhancement of muscle growth is one of the most desirable effects in sport doping. In this context, MYO inhibition/suppression sounds as a very effective strategy for the non-physiological, illicit improvement of sport performance, especially in power sports/sport disciplines. For the above reasons, anti-MYO agents are included in the list of doping substances and methods of the World Anti-Doping Agency (WADA): section S4 (“Hormone and metabolic modulators”) specifically mentions as a doping strategy the use of “MYO inhibitors such as i) Agents reducing or ablating MYO expression, ii) MYO binding proteins (such as FOL, and MYO propeptide) and iii) MYO-neutralizing antibodies” [4]. No direct methods are currently available for the detection of all the many possible substances and methods triggering MYO inhibition.

Due to the wide variety of substances and methods that can be used to inhibit/suppress MYO, the detection of doping by MYO inhibition represents one of the most demanding challenges for the anti-doping scientific community. At present, there are no known official methods for the detection in serum/urine of antibodies neutralizing MYO, but, even though these methods were available, they would be useful only for detecting a small part of the different possible inhibition/suppression systems. For instance, a method specifically developed for the detection of anti-MYO antibodies would have no efficacy in the case the inhibition would be performed using FOL and/or gene therapy with siRNAs.

Nowadays, skeletal muscle is considered as an endocrine organ secreting hormone-like factors that can influence the metabolism of other organs and tissues. During muscle contraction, the production and secretion of specific myokines are stimulated, and myokines can act with endocrine, autocrine and/or paracrine action. In addition to MYO and to its primary antagonist FOL [5], other myokines of interest are the following: the adipomyokine Follistatin-like 1 [6] (FSTL-1), Musclin (MUS) [7], Oncostatin (ONC) [8], Osteonectin (OST) [9], Irisin (IRI) [10], Brain Derived Neurotrophic Factor (BDNF) [11], Insulin-like Growth Factor 1 (IGF1) [12], some interleukins such as IL-6, IL-13, IL-15 [13] and some other growth factors such as Fibroblast Growth Factor-21 (FGF-21) [14] and also Erythropoietin (EPO) [15]. The activity of all these myokines is related to the trophic state and to the activity of the skeletal muscle; moreover, their action is often synergistic.

The primary way to inhibit MYO is by the use of FOL – a ubiquitous glycoprotein produced by the anterior pituitary gland [16], that, consequently, cannot be classified as a myokine – and/or of antibodies binding to MYO itself or to the ActRIIB receptor. Recently, a spliced form of FOL was produced and successfully delivered into muscles by using adeno-associated viruses (AAV) with no appearance of adverse effects [17]. Other methods that can be illicitly used to inhibit MYO are based on the silencing of the MYO gene by gene therapy (GT), via the transfer of genetic material (RNA, DNA, or genetically modified cells) to human cells, normally with the purpose to treat specific diseases [18]. It was also shown that in vivo injections of MYO binding antibodies can be effective in neutralizing MYO as a therapeutic strategy to treat dystrophies [19]. In recent years RNA interference (RNAi) has also been proven to achieve MYO inhibition. More specifically, RNAi uses small RNA molecules, called short interfering RNA (siRNA), that bind to mRNA and promote post-transcriptional gene silencing [20]. A recent study also reported that the use of antisense oligonucleotides can be effective in MYO blockade and that the inhibition can be achieved also by exon skipping [21]. Finally, apart from siRNA, other possibilities to inhibit MYO gene expression are represented by microRNAs (miRs), that are small endogenous non-coding single strand RNAs whose function is to regulate gene expression in an epigenetic way [22]. In particular, miRs induce gene silencing by overlapping with complementary sequences present on the mRNA target molecule; this link entails a repression of the translation or the degradation of the target molecule at a post-transcriptional level. Several miRs, called MyomiRs, are expressed in muscle cells where they act by repressing several genes responsible for the growth and development of the muscle cells themselves. Their expression is cell-and tissue-specific, and their role as cellular biomarkers in the diagnosis of pathologies is progressively being explored. The majority of miRs are present within the cell, but they have also been found in significant levels also extracellularly, in many biological fluids, such as plasma, serum, saliva, and urine.

Such a broad variety of possible ways to reduce the levels of MYO make the development of traditional, “direct” antidoping analytical methods, aimed to detect all the potential MYO inhibitors/suppressors, virtually impossible. In cases like this, the only possible detection strategy is based on the monitoring of a panel of diagnostic bio-markers, whose levels are significantly altered following MYO inhibition/suppression. The basic idea of the present work is therefore to identify possible biomarkers that can be perturbed by the MYO suppression. We focused on protein targets (not only MYO itself and FOL, but also some other myokines closely related to MYO) and on their possible correlation. Since the main aim of the study was to design a procedure that could be applied in the framework of doping control, our first step was to assess the normal physiological levels of the selected biomarker(s) and the possible correlation among the targeted biomarkers themselves in elite athletes testing negative to the antidoping tests. The analysis of these data would indeed represent the first step in the aim to detect a “signature” of the abuse of the inhibition/suppression of MYO, whenever the normal values are perturbed by non-physiological causes. If proved sufficiently robust, multi-parametric assays of this kind can be taken into consideration as effective initial testing methods.

On the grounds stated above, we have therefore considered the following:

1. To explore the potential application of human serum myokines as indirect markers of doping and principally to define ranges of baseline serum levels of the principal myokines in a database of elite athletes.
2. To evaluate the correlation among the myokines tested and their variability considering sex, age and sport/discipline.

## MATERIALS AND METHODS

### Participants

Serum samples from elite athletes (see Table 1 for details on the composition of the selected athlete population) collected for routine doping control tests and previously reported as negative have been assayed for the selected panel of following, potential biomarkers: Myostatin (MYO); Follistatin-like 1 (FSTL-1); Musclin/Osteocrin (MUS); Oncostatin (ONC); Osteonectin (OST); Irisin (IRI); Brain-derived Neurotrophic Factor (BDNF); Follistatin (FOL); Insulin-like growth factor 1 (IGF1), in compliance with the World Anti-Doping Agency ethical code for the accredited laboratories [23]. All samples considered in the present study were from athletes who explicitly expressed their consent for the samples to be used for research purposes on the doping control form, after anonymization and after all anti-doping analyses were completed, with no adverse analytical finding reported for any other doping substance/method. The information reported on the doping control form, indicating sex and age of the subject, allowed to exclude that more than one sample among those considered in the study was collected from the same subject. Athletes were divided into sub-groups according to their sex and to the type of sport to which they belong. Athletes practicing POW sports were selected among disciplines such as weightlifting, body building, short-distance athletics (less than 200 meters). Athletes practicing endurance sports (END) were selected among disciplines such as cycling, football, tennis, triathlon, long-distance athletics (more than 1000 meters).

**TABLE 1:** Details on the elite athletes’ population selected for the construction of the database and immunological assays applied. Pow = Power sport, End = Endurance sport, FCBA = Flow cytometry bead array, IRMA = Immunoradiometric assay, ELISA = Enzyme-Linked Immunosorbent Assay, I-125 = Iodine isotope 125; LOD = limit of detection.

	All	Age mean	Males	Females	Pow	End	Assay	Detection	LOD	Precision	

		(min-max)					Test			Intra-assay	Inter-Assay
**BDNF**	56	26.6 (17–46)	43	13	18	38	FCBA	Fluorescence	6 pg/mL	< 10%	< 15%
**FSTL-1**	58	26.7 (17–46)	45	13	19	39	FCBA	Fluorescence	296 pg/mL	< 10%	< 15%
**IRI**	41	26.9 (17–46)	35	6	16	25	FCBA	Fluorescence	150 pg/mL	< 10%	< 15%
**MUS**	51	26.8 (17–46)	39	12	15	36	FCBA	Fluorescence	37 pg/mL	< 10%	< 15%
**MYO**	53	26.6 (17–46)	41	12	18	35	ELISA	Absorbance	5.3 pg/mL	< 5%	< 10%
**ONC**	58	26.8 (17–46)	45	13	19	39	FCBA	Fluorescence	0,4 pg/mL	< 10%	< 15%
**OST**	55	26.8 (17–46)	44	11	18	37	FCBA	Fluorescence	2.6 ng/mL	< 10%	< 15%
**IGF1**	57	26.7 (17.46)	44	13	19	38	IRMA	I-125 decay count	10 ng/mL	< 10%	< 10%
**FOL**	54	26.8 (17–46)	42	12	19	35	ELISA	Absorbance	83 pg/mL	< 5%	< 10%

### Immunological Assays

Different immunological methods with antibodies specific for the proteins of interest were used. More specifically, MYO and FOL levels were measured by enzyme-linked immunosorbent assay (ELISA, R&D Systems, article # DGDF80 and DFN00 respectively) on a Victor3 plate reader (Perkin Elmer) using quality controls prepared by the recombinant reference standards included in the respective kits; IRI and IGF1 serum levels were measured by radioimmunoassays (RIA, Phoenix Pharmaceuticals) on a Wizard2 Gamma Counter (Perkin Elmer); the analysis of all other analytes was performed via multiplex cytometric beads array (CBA, Merck Millipore) on xMAP Luminex 200 Flow Cytometer (Merck Millipore). Samples were assayed in duplicate and results were considered valid when intra-day precision was estimated to be less than 10% of coefficient of variation.

Table 1 summarizes the characteristics of the athletes and the immunological assays applied in this research.

### Data Analysis

Descriptive statistics was performed by using Statistica 12.0 (Statsoft) and SPSS 17.0 (IBM) software packages. Kolmogorov-Smirnov test was used to assess the normality of data distribution. Correlations among biomarkers were calculated according to Kendall’s tau beta index. Differences between groups have been determined by Mann-Whitney U test except where indicated. Statistical significance was set at p < 0.05, high significance was considered when p < 0.01. A Principal Component Analysis (PCA) was run for a reduced and paired subset of the database (N = 41) in which all athletes resulted assayed for all the analytes.

### Ethics

The authors declare that the experiments reported in the manuscript were performed in accordance with the ethical standards of the Helsinki Declaration and that the participants signed an informed consent form. Specifically, anonymized samples were selected among those belonging to athletes who had given written consent for their samples to be used for research purposes at the moment of sample collection for the doping control test, in agreement with the WADA Ethical rules as detailed in the WADA International Standards for Laboratories.

## RESULTS

Table 2 summarizes the results of the assays of myokines in elite athletes’ serum.

**TABLE 2:** Serum level of Myostatin (MYO), Follistatin (FOL), Insulin-like Growth Factor 1(IGF1), Oncostatin (ONC), Osteonecton (OST), Follistatin-like-1 (FSTL-1), Musclin (MUS), Irisin (IRI), Brain Derived Growth Factor (BDNF) in elite athletes and values comparison among groups of interest. M = males, F = females, POW = power typically anaerobic sports, END = endurance typically aerobic sports. * = significance (p < 0.05), ** = highly significant (p < 0.01).

		N	mean (ng/mL)	std.dev	std.err.mean	*p*-value
**BDNF**	ALL	56	10.32	8.71	1.16	
	M	43	10.14	8.66	1.32	0.99
	F	13	10.92	9.22	2.56	
	POW	18	15.75	1.10	2.59	0.001^**^
	END	38	7.75	6.03	0.98	

**FSLT-1**	ALL	58	27.35	18.26	2.40	
	M	45	24.26	15.44	2.301	0.014^*^
	F	13	38.01	23.46	6.506	
	POW	19	28.45	1.582	3.629	0.58
	END	39	26.81	1.951	3.123	

**IRI**	ALL	41	1.62	1.56	0.24	
	M	35	1.424	1.191	0.201	0.43
	F	6	2.736	2.83	1.155	
	POW	16	1.831	1.971	0.493	0.86
	END	25	1.479	1.251	0.25	

**MUS**	ALL	51	0.320	0.19	0.03	
	M	39	0.293	0.199	0.032	0.03^*^
	F	12	0.395	0.117	0.034	
	POW	15	0.333	0.185	0.047	0.76
	END	36	0.309	0.19	0.032	

**MYO**	ALL	53	3.85	1.89	0.26	
	M	41	3.871	1.883	0.294	0.39
	F	12	4.345	1.912	0.552	
	POW	18	4.332	2.683	0.632	0.69
	END	35	3.796	1.309	0.221	

**ONC**	ALL	58	0.061	0.14	0.02	
	M	45	0.068	0.16	0.024	0.20
	F	13	0.036	0.013	0.004	
	POW	19	0.045	0.04	0.019	0.47
	END	39	0.069	0.171	0.027	

**OST**	ALL	55	0.42	0.24	0.03	
	M	44	0.397	0.233	0.035	0.25
	F	11	0.534	0.262	0.079	
	POW	18	0.441	0.215	0.051	0.49
	END	37	0.417	0.258	0.042	

**IGF1**	ALL	57	197.3	73.6	9.744	
	M	44	193.9	76.7	11.568	0.30
	F	13	208.5	63.1	17.491	
	POW	19	236.7	67.608	15.51	0.006^**^
	END	38	177.6	69.058	11.203	

**FOL**	ALL	54	1.37	1.21	0.09	
	M	42	1.127	0.587	0.09	0.33
	F	12	1.165	0.918	0.265	
	POW	19	1.022	0.319	0.073	0.80
	END	35	1.196	0.789	0.133	

**FIG. 1:**
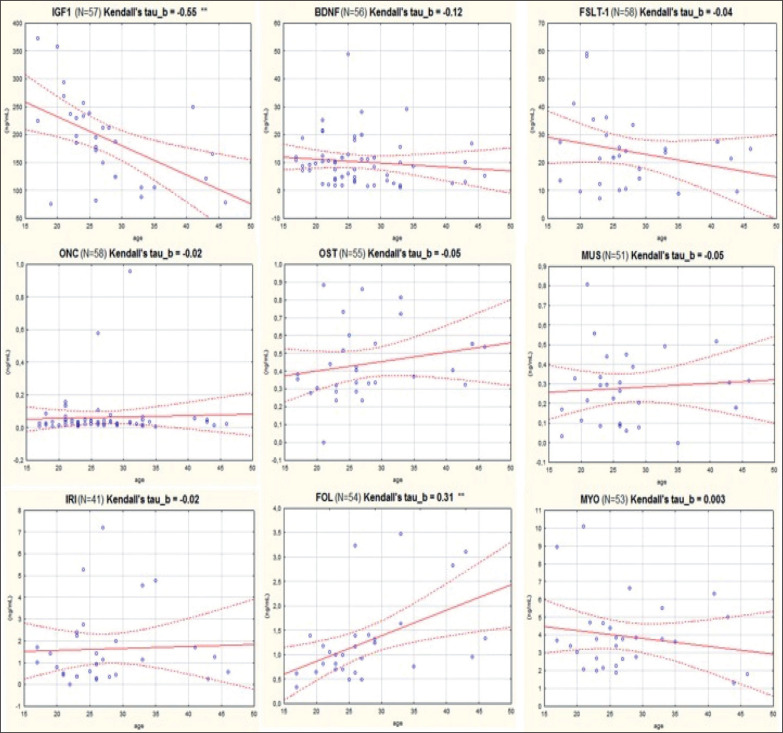
Correlation with age of the levels of each of nine biomarkers considered in this study. IGF1: Insulin-like growth factor 1; BDNF: Brain-derived Neurotrophic Factor; FSTL-1: Follistatin-like 1; ONC: Oncostatin; OST: Osteonectin; MUS: Musclin/Osteocrin; IRI: Irisin; FOL: Follistatin; MYO: Myostatin.

As it can be seen, serum levels varied by different orders of magnitude among the different target compounds: ONC and MUS showed concentrations lower than 1 ng/mL; levels of FST, MYO and IRI ranged between 1 and 5 ng/mL, while BDNF, FSTL-1, IGF1 and were highly expressed in serum (BDNF and FSTL-1 approximately between 10 and 30 ng/mL and IGF1 approximately 200 ng/mL). A statistically significant difference was found between female and male subjects regarding FSTL-1 and MUS, where female athletes showed higher values of these analytes. Moreover, another statistically significant difference was found in the levels of IGF1 depending on the type of sport practiced by the athletes: subjects belonging to the POW group presented significantly higher serum values of IGF1. The serum concentration of MYO resulted 4 to 5 times higher than FOL. Differently, FSTL-1 presented higher average serum values than MYO and the ratio in all samples resulted greater than 1. Figure 1 shows the correlation plots of the analytes versus the athletes’ age. As it can be seen, negative and statistically significant correlation was found only for IGF1 (Kendall’s tau beta index of correlation = -0.55, p < 0.01).

**FIG. 2:**
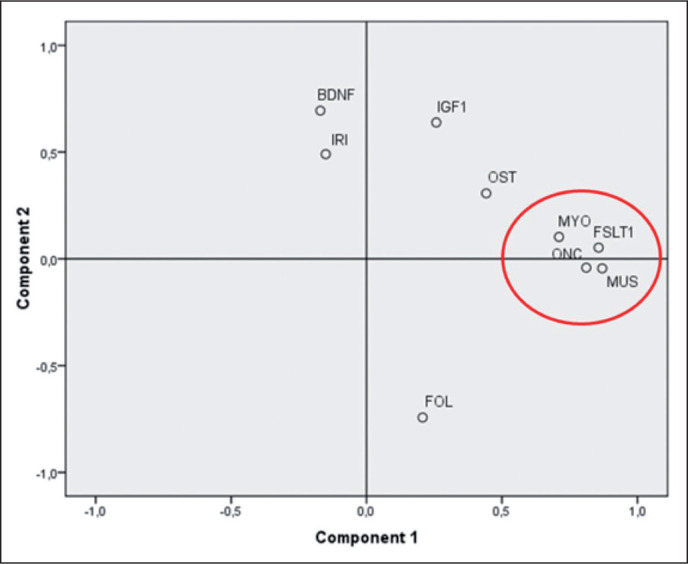
Principal component analysis of a paired subset of athletes (N=41). Component #1 explain 33% of the total variance and can be related to the development and trophy of muscles and bones. Component #2 explain another 17% of the variance and can be interpreted as the component mainly related to the energetic metabolism of the skeletal muscle. IGF1: Insulin-like growth factor 1; BDNF: Brain-derived Neurotrophic Factor; FSTL-1: Follistatin-like 1; ONC: Oncostatin; OST: Osteonectin; MUS: Musclin/ Osteocrin; IRI: Irisin; FOL: Follistatin; MYO: Myostatin.

A weak, but significant, positive correlation was found for FOL (Kendall’s tau beta index of correlation = 0.31, p < 0.01). PCA analysis (figure 2) showed that three myokines, MUS, FSTL-1 and ONC, are more related to MYO as they cluster very closely together in the first component of the plot.

## DISCUSSION

### Myokines in Athletes Serum: Descriptive Statistics

All myokines analyzed resulted to be measurable in all samples of human serum, except for MUS and IRI, whose concentrations were in some samples below the limits of quantification (0.037 and 0.15 ng/mL respectively). Myokines showed levels of serum concentration of different orders in magnitude: OST and MUS resulted to have a concentration lower than 1 ng/mL; FOL, MYO and IRI between 1 and 5 ng/mL, while BDNF, FSTL-1 and IGF1 were highly expressed in serum with concentration higher than 10 ng/mL.

For FSTL-1 and MUS, females exhibited higher serum values than males (p < 0.05). However, the overall limited number of female individuals in the database may be a confounder of this result.

A statistically significant difference was also found considering the type of sport practiced by the athletes (POW vs. END) with regard to IGF1. POW group presented significantly higher serum values of IGF1. The reasons for this result can be found in the peculiar characteristics of strength power sport types. POW athletes perform high intensity performances in a very short time range, and which exploit an anaerobic alactacid energy metabolism by using phosphocreatine as the main substrate for ATP regeneration. IGF1 is a growth factor that is secreted in response to the stimulation of growth hormone which in turn is under the control of the hypothalamus. IGF1 is a growth factor essential for development and growth of tissues and bones. Despite skeletal muscle is not the main secretory organ, IGF1 has been recognized recently as a myokine to all extents [24].

Acute high intensity exercise is a very powerful stimulus for GH secretion, which in turn can therefore lead to increased IGF1 production hence the higher serum values found in the power group. In any case, the true relationship between IGF-1 and physical exercise is still to be fully understood. Other studies report contradictory results where certainly the IGF1 increases following a high intensity exercise but while a low intensity exercise would lead to the maintenance or even the decrease of the serum values of IGF1 [25].

### FOL/MYO and MYO/FSTL-1 Ratios

On average, the serum concentration of MYO resulted to be 4 to 5 times higher than FOL. The ratio between the two analytes has a wide, non-normal distribution and resulted lower than 1 with a slightly asymmetric distribution (mean value = 0.34). A reversal of the FOL/MYO ratio could indicate an inhibition of MYO through the fraudulent use of its natural antagonist(s). However, the high variability we found for the ratio (CV = 64%), suggests that it may be difficult to fix a population threshold of abuse in order to use the ratio as an index of MYO inhibition. For antidoping purposes, it may be more appropriate to follow the variation of individual MYO/FOL ratio longitudinally. A similar consideration can be made with respect to the MYO/FSTL-1 ratio. In this case, FSTL-1 exhibits higher average serum values than MYO and the ratio in all samples is greater than 1. The alteration of the ratio to values close to 1 or even below 1 could be caused by the inhibition of MYO through an over-expression of its inhibitor FSTL-1. However, also in this case, we found a very wide distribution of the ratio values (mean ratio = 7.8, CV = 85.4%) making it difficult to establish population-based thresholds.

### Correlation with Age

The decrease in serum IGF-1 with age is well a known phenomenon [26–27]. With respect to sport doping, IGF-1 is not only prohibited as such, but it is also one of the two biomarkers to detect GH intake: the age of the subject is taken into account in the GH-Score formula applied in doping control to determine the decision limit of the abuse [28].

Our data are in agreement with the above: a negative and statistically significant correlation with age was found for IGF1 (Kendall’s tau beta index of correlation = -0.55, p < 0.01, see Figure 1).

On the other hand, another statistically significant correlation with age was found for FOL (Kendall’s tau beta index of correlation = 0.31, p < 0.01), although this correlation seems to be weak as indicated by the index of correlation that is < of 0.5. A more detailed analysis was performed by dividing the athletes into two groups of equal numbers (up to 25 years of age N = 29, and from 25 years upwards N = 28). Athletes over 25 years of age have higher FOL (1.42 ng / mL vs. 0.85 ng / mL) and also in this case the result obtained is statistically significant (p < < 0.01). FOL actually increases with age and this result is confirmed in observational studies from literature: mean FOL levels do not change during puberty, but are higher in adult and postmenopausal women [29]. On the other hand, we found no correlation between age and MYO in our database. From the literature, it is known that MYO slightly increases until 57 years of age and then it starts to decrease [30]. Moreover, some studies found that serum MYO levels follow a circannual cycle, showing a peak during the spring season, as they are directly dependent on the 25-hydroxy-Vitamin D levels [31]. It was also found that females have slightly lower serum levels of MYO than men [32]. However, in our analysis we did not find such differences: we found MYO values to be moderately higher in females, but without being statistically significant and this result may also be due to the small number of female athletes in our database. Moreover, the fact that FOL shows a positive correlation with the age of athletes while on the contrary MYO does not (at least in the age range considered in this study), confirms that any monitoring of the MYO / FOL ratio for anti-doping purposes would perform better if executed at the longitudinal level by applying indi-vidual thresholds of abuse.

### Correlation among Myokines

Correlation among myokines was assessed by calculating Kendall’s Tau beta index of and by performing a Principal Component analysis (PCA) (see again Figure 2). PCA analysis shows that three myokines, MUS, FSTL-1 and ONC, are closely related to MYO. These myokines cluster very closely together in the first component of the plot. This component can be interpreted as the one associated to the development of skeletal muscle mass and its state of trophy. The second component (explaining another 20% of the total variance) can be interpreted as linked to the trophic and energetic/anabolic state of muscle tissue, where analytes such as BDNF, IRI and IGF1 show the highest values. PCA results are corroborated by the significance that emerged from the calculation of the correlation indices as described in Table 3. The pairwise correlations between ONC, MUS and FSTL-1 are high and highly significant (p < < 0.01). MYO correlates moderately but significantly with FSTL-1 and ONC (p < 0.05) and more weakly with MUS. The close proximity of FSTL-1, MUS and ONC to MYO can be explained the physiological features of these three myokines. FSTL-1 is, like myostatin, a myokine that accentuates hypertrophy [33], specifically of the cardiac muscle and improves regeneration after heart attacks. It belongs to the Osteonectin protein family also known as SPARC (Secreted Protein Acidic and Rich in Cysteine). It is known that FSTL-1 is principally secreted by skeletal muscle cells, it is induced in skeletal and cardiac muscle by hypertrophic response and it has a protective effect on muscle vasculature [34]. Most important of all, FSTL-1 it is a MYO inhibitor as well as FOL [35].

**TABLE 3:** Correlations among myokines and the other analytes calculated by Kendall’s tau beta index. BDNF: Brain-derived Neurotrophic Factor; FSTL-1: Follistatin-like 1; ONC: Oncostatin; OST: Osteonectin; MUS: Musclin/Osteocrin; IRI: Irisin; FOL: Follistatin; MYO: Myostatin.

	BDNF	FSLT-1	ONC	OST	MUS	IRI	FOL	MYO
**BDNF**	-							
**FSLT-1**	0,05	-						
**ONC**	0,09	**0,46** ^**^	-					
**OST**	-0,05	0,11	0,03	-				
**MUS**	0,09	0,50^**^	0,56^**^	0,19	-			
**IRI**	0,20	-0,15	-0,23	0,21	-0,14	-		
**FOL**	-0,17	0,05	0,13	-0,02	0,10	-0,21	-	
**MYO**	-0,08	0,17	0,23^*^	0,04	0,13	-0,10	-0,05	-

* = significance (p < 0.05),

** = highly significant (p < 0.01).

ONC promotes bone formation [36]. Levels of ONC, which was initially identified in the bone, increase during muscle development and regeneration but also during en-durance exercise and muscle hypertrophy. The close proximity between ONC and MYO is not surprising when we consider that MYO itself is involved in the formation of osteoclasts and bone resorption [37]. MUS is an exercise responsive myokine that is known to improve exercise capacity and physical endurance [7]. Apart from the properties and functions that each of these myokines possesses, their correlation with myostatin suggests that inhibition of myostatin (evidenced by a reduction in its serum levels) may also affect the serum values of these three related-myokines. This circumstance is of considerable importance in the view to consider the myokines as effect biomarkers for anti-doping purposes.

## CONCLUSIONS

In this pilot study, we examined a pool of human myokines to assess their variability in serum samples of professional athletes and their potential feasibility as biomarkers to reveal the manipulation of muscle cells regulators for doping purposes.Results can be summarized as follows:

– All myokines were assayable, although their levels in human serum varied by different magnitude in concentrations. Due to the limited availability of studies concerning specific myokines in sports, the concentration values of the myokines reported here can serve as a starting point to assess reference ranges by further studies, conducted on a broader population of athletes.
– MYO serum concentration was found to be higher than FOL concentration. The MYO/FOL ratio showed a wide distribution that does not suggest its application to doping control analysis as a diagnostic paremeter for Myostatin inhibition. Individual and longitudinal-based follow up of the MYO/FOL ratio, as in the frame-work of the Athlete Biological Passport, also needs to be explored.
– A group of three myokines (MUS, FSTL-1 and ONC) clusters very close together with myostatin. This correlation makes these three myokines to be good candidates as potential biomarkers of inhibition practices operated on myostatin. Further studies, made also on a greater number of athletes and treated with myostatin inhibitors will further clarify the real extent of this correlation.

Results here obtained suggest that, although muscle damage due to very intense training could lead to a leakage of myokines into serum, the identification of a range of baseline levels, especially for MYO and for four of the target analytes here considered (FOL, FSTL-1, MUS and ONC), with the possibility of monitoring their variation longitudinally – as in the framework of the “Athlete biological Passport” – also following additional studies on their intra-individual variability, may be the starting point to develop a novel strategy to reveal the different forms of doping based on MYO inhibition/suppression.

## Data Availability

The data that support the findings of this study are available on request from the corresponding author. The data are not publicly available due to privacy or ethical restrictions.
